# Collectanea Medica

**Published:** 1814-11

**Authors:** 


### COLLECTANEA MEDICA.

*Case of Tænia in an Infant, cured by Decoction of Pomegranate.* By WILLIAM POLLOCK, M.D. Communicated by ADAM BURT, M.D. Superintending Surgeon, Bengal.

I FORMERLY communicated to you some cases of tænia, cured by the pomegranate decoction, which were inserted by Dr. Fleming in his Catalogue of Indian Medicinal Plants and Drugs, printed in 1810, and, since that period, I have found the remedy invariably successful, in a very great number of instances. In many of those the tænia had acquired an enormous length, and in some of them it was received in tepid water, and lived for several hours after it was passed. The following case occurring in an infant, not two months weaned, appeared to me to be very remarkable, in consequence of which I reported it to you, and also sent a copy of it to Dr. Fleming, at the time it happened. That it is a very uncommon occurrence, may be inferred from an observation of Dr. Hamilton's, in his most valuable work on purgative medicines, where he says, that the tænia is altogether unknown in infancy and childhood.

Peter Daly, aged fourteen months. 27th August, 1811. Was weaned about two months ago, and has since been gradually drooping, in consequence, as was supposed, of dentition. He is now excessively reduced, refuses all food, and is harassed with a constant troublesome diarrhœa. His skin is loose, dry, and shrivelled, and he has the whining fretful cry of a child who has been long sick. Different medicines have been prescribed for this diarrhœa without relief, and for several days his stools have contained small fleshy shreds, some of which considerably resemble half-dissolved portions of the tape-worm. Two ounces of water were added to six of a decoction of the pomegranate root, (prepared by boiling two ounces of its fresh bark in a pound of water to nine ounces,) and a table spoonful was ordered to be given the child every half hour, unless sickness and vomiting intervened.

28th.—He took the whole of his medicine yesterday, without either sickness or vomiting, and in the evening he passed a portion of tape-worm alive, upwards of six feet long. The medicine purged him briskly, and to-day he has vomited almost every thing he has taken. Appearing much exhausted, an anodyne carminative mixture was ordered to be given at intervals, to relieve the sickness.

29th.—Appears more lively; has had no vomiting since yesterday, and the diarrhœa has been much restrained by the anodyne. Quiescat.

1st September.—Is manifestly better in every respect, but his stools still contain portions of the tape-worm. Eight ounces of the above decoction, without dilution, were ordered to be given in the same manner as before.

2d.—He took the whole of his medicine yesterday, without either sickness or vomiting, and with but little effect upon his bowels, until this morning, when it began to purge him briskly, and he passed another portion of the tape-worm, nearly eight feet long. He has been very hungry, and ate a hearty breakfast.

25th.—No portions of the tape-worm have been observed since last report, and the diarrhœa gradually left him, without the use of any other medicine. His bowels have become regular, his appetite keen, and he has filled up apace. He has the appearance of a healthy thriving child, and his strength has improved so rapidly, that he has now begun to walk.

From the above period he continued to thrive until August 1812, when when he again began to pass pieces of the tape-worm. The pomegranate decoction was repeated, and he passed an entire tænia alive, fifteen feet long, since which he has been in perfect health, and is at this moment a very fine boy. He is son of Edward Daly, private soldier in his Majesty's 53d regiment.—*Edin. Med. and Surg. Journal.*

---

*Observations on Pemphigus.* By Dr. DICKSON, Physician and Inspector of the Fleet, &c. on the North-American Station.

CASE I.—G. Michaloff, ætatis 56, belonging to the Russian fleet, was admitted into the Argonaut, hospital-ship, on the 19th November, 1813, in consequence of a vesicular eruption, of three days standing, which occupied almost every part of the body, but particularly the face, arms, and thighs. The vesicles were largest upon the thighs; but they were of every size, from a pea to that of a walnut, irregularly circumscribed, but generally oblong, and filled with a straw-coloured transparent fluid. Some of them were broken, and had discharged a large quantity of serum. The pulse was 110, the heat somewhat increased, but the tongue moist and clean. He was directed to take a purgative, and to be put into the tepid bath. To avoid repetition, I shall confine my observations chiefly to the progress of the eruption, and content myself with remarking generally, that the febrile symptoms were mild, and the functions nearly natural; and that the only treatment indicated throughout the disease consisted of the occasional exhibition of a purgative, and some saline antimonial medicine, with a wash for his eyes; and mild dressings, after bathing the excoriated parts in tepid water.

20th.—Many of the old vesicles are broken, and several new ones have appeared. There are some of a small size upon the eye-lids and tarsi, and the eye-lids are inflamed. He complains only of the soreness of the excoriated parts; appetite and spirits good.

21st.—The vesicles on the eye-lids are ruptured; the tunica conjunctiva is much inflamed, and the exposure to light causes a copious flow of scalding tears.

22d.—As the old eruptions burst, new ones appear in various parts of the body. The ophthalmy has increased, the tarsi are swollen and ulcerated, and there is a copious agglutinating discharge which prevents the opening of the eye-lids, until bathed with milk and water.

23d.—Few new vesicles have occurred since yesterday. He feels very sore; and the excoriated places secrete a copious purulent discharge.

24th.—The eye-lids are closed and swollen, and there is a considerable puriform discharge; but no farther vesications have appeared. After this date no new vesicles formed; and the sores, after discharging considerably, dried up by degrees, leaving the skin of a deep red colour. On the 1st January, 1814, he was discharged; but was sent back again to the hospital-ship in about a fortnight fortnight, in consequence of several small blisters having risen on the scrotum and penis. They were not, however, larger than a pea, and soon healed; and, no new vesicles having appeared on any other part, he was, after some time, finally dismissed. The skin retained a mottled appearance, from deep red patches of various dimensions.

He was not sensible of any indisposition before the eruption took place. He stated that he had been affected with the same complaint sixteen years ago, but was not aware of any cause, nor of having seen any one else with the same disease.

CASE II.—J. Plusnin, ætatis 39, also a Russian, of a spare habit, was received into the Argonaut, on the 23d of November, 1813, labouring under fever, from which he was nearly recovered, when, on the 1st of January, 1814, some small vesicles were observed at the bend of the arm, where venesection had been performed. The pulse, skin, and tongue, were nearly natural.

2d.—He yesterday took a purgative. Some eruptions have appeared farther up the arm, and upon other parts of the body, which, on breaking, discharge a lemon-coloured serum.

6th.—Since the 2d, vesicles have continued to appear, and they are now general over the body, and upon the head, where they are very large. On the body, though most frequently the magnitude of a nut, they are of every size, from a pea to a walnut. The eyes, as in the case preceding, are much inflamed.

10th.—The hairy scalp is almost covered with large bullæ, which, bursting, discharge an astonishing quantity of glutinous fluid, forming, when dry, an incrustation very difficult to remove. There are also many small blisters in the mouth, which give great pain; particularly those on the edges, and under the tongue. He cannot bear the light for an instant; and the attempt to open the eye-lids causes a copious flood of tears, by the acrimony of which the tarsi and cheeks are red and excoriated. The pulse, however, is only 85.

11th.—No fresh eruptions have appeared; many of the older vesications are dried, and covered with an incrustation; but others still discharge copiously, particularly those on the scalp, from which it is so profuse as to wet his night-cap and pillow-case in a very short time; the edges of the tongue are much ulcerated.

12th.—He feels very sore, and the excoriations are so general and extensive as to make every position irksome. He cannot allow his eyes to be inspected. The pulse is 100, and he is low; but takes some arrow-root and wine.

14th.—There were no new vesicles yesterday, but three have since appeared on the neck. The pulse is 80 in the morning, but considerably quicker in the evening.

15th.—No farther eruption; but the general irritation and soreness are very distressing, and the discharge continues copious, particularly about the joints, and from the parts upon which he presses; the eyelids are closed, ulcerated, and much swelled.

16th P.M.—The pulse rises in the evening at 110, and he feels very very sore and feeble; but no fresh vesications have arisen. From this period that the eruption ceased, his health and strength declined; and the exacerbations in the evening increased. It was vainly attempted to support his strength by a more nourishing diet, wine, a decoction of bark, and by opiates to procure rest. Notwithstanding every attention paid to his unfortunate case by Dr. Douglas, the surgeon of the hospital-ship, he became every day more feeble and emaciated, and died on the 29th of January, perfectly exhausted and worn out by the sufferings caused by the irritation, soreness, and discharge. Although some marks of disease were discovered on dissection, and particularly in the thorax, yet the viscera in general were much sounder than they were usually found in those who had died of fever, or its consequences. The small intestines, however, contained a yellow, glairy, gelatinous-looking matter; and on their outer surface several yellow patches were observed; but it was difficult to determine whether these depended on an exudation of the same fluid between the coats, or upon the peritonæum having been elevated so as to have formed distinct vesicles at an earlier period of the disease. No vesicles were discovered on the inner surface of the intestines, although in some places it looked redder than natural. The contents of the right eye were found destroyed by an abscess, and the cornea of the left was opake and ulcerated; the arm-pits, groins, scrotum, and between the fingers, buttocks, &c. were perfectly raw, like a blistered part; and in other places the skin appeared dried and puckered up, and of a deep purple colour.

Of the third case, likewise a Russian, I did not preserve any account, as fever prevailed to a great extent at the time; and it was divested of interest, by nearly all the vesicles having been ruptured before his admission into the Trusty, hospital-ship. They had been numerous, but were not succeeded by ulceration nor any constitutional symptoms worthy of notice; and he was well again in about a fortnight; the exudation from the vesicated parts generally forming a rough disagreeable brownish incrustation, which gradually peeled off.

The fourth case I did not see, as it occurred between the two periods of sickness, during which I was superintending physician of the Imperial Russian fleet; but I have been favoured with some information respecting it by Mr. Dobson, surgeon of the Trusty, hospital-ship, which I shall give nearly in his own words:

Jacques Jejaurnoy, a French prisoner, ætatis 48, was admitted on the 25th of April, 1813, labouring under cough, purulent expectoration, hectic sweats, and other symptoms of confirmed phthisis pulmonalis. In the night of the 5th of May, after a profuse perspiration, he was annoyed by a troublesome itching of his thighs; and, on putting down his hand to scratch them, he felt several elevations of the cuticle, about the size of a large pea, but paid no farther attention to them at the time. The itching returned the next night, and more vesicles formed; and, on the following morning, the first vesicle broke, as the patient asserted, with an audible audible noise. After this period some rose on his left side; and, on the evening of the 9th, one made its appearance on the left cheek. They continued to form and to burst in various places successively until the 16th of May; subsequently to which date no fresh blebs appeared, though some of the former did not break for several days after that period. The largest, which was upon the thigh, was, on the 14th, about the size of a large nutmeg; on the 15th it had become elongated, and measured an inch and three quarters in length, and about an inch in breadth. It did not appear to be altogether filled with serum; but, as it recovered its elevation, after pressure, seemed to contain a small portion of a gazeous fluid; a circumstance which, if my recollection be not erroneous, is noticed by some writer, and which is agreeable to the etymology of pemphigus.

The appearance of the eruption, as in the second case, seems to have been somewhat critical; for Mr. Dobson mentions, that the man's health and appetite improved so much, and the phthisical symptoms were so far suspended for a time, that he felt a strong inclination to inoculate some other consumptive patients with the fluid, but was deterred by some accounts of the formidable nature of this disease. A smarting sore followed the bursting of the vesications; but there was not any inflammation round their bases; nor did they leave any pits, as they generally became dry in two or three days, and terminated in a brownish-coloured desquamation.

A French assistant-surgeon, who was then in the ship, stated that he tasted the fluid, which was of an acid quality; and that it turned the red threads of the rug upon the bed of the patient, upon which it fell, to a dirty brown colour.—*Edin. Med. and Surg. Journal.*

---

*Memoir upon the compound and smooth or simple Eyes of Insects, and on the Manner in which these two Species of Eyes concur in Vision.* By M. Marcel de Serres, Professor of the Sciences in the Imperial University.

(*Continued from p. 322.*)

The choroid of the compound eyes of insects has been regarded by Swammerdam as an uvea. It would seem, however, that it cannot be called an uvea, as it does not occupy the bottom of the eye of insects. However this may be, this membrane is attached by its circumference to the whole of the edge of the cornea, and consequently follows the contours of this same membrane. It is surrounded by a large circular trachea, in general furnished by the tracheal artery of Swammerdam, but varying as to its arrangement in the different genera. For instance, in the *gryllus* and *truxalis*, it is the third principal division of this same trachea situated in the head, which, when it arrives at the eye, becomes bifurcated, and the two tracheæ which result fasten on the edges of the eye: the latter form the large circular trachea, unite afterwards to the upper part, part, continuing after this junction in one single trachea, which, by joining to another, afterwards terminates at the base of the brain.

The large circular trachea furnishes an infinite number of very minute tracheæ, which soon becoming bifurcated, form very numerous isosceles triangles which rest on the circumference of the optic cone. These triangles formed by the tracheæ are divided as by a perpendicular, by the nervous filaments resulting from the expansion of the optic nerve. These filaments afterwards pass through the choroid and its opaque varnish, as well as the tunic of the cornea, and terminate below the facets of the last-mentioned membrane. This arrangement of the tracheæ, and of the nervous filaments, forms a handsome network, which is rendered very sensible by carrying the nerve inwards, and on the side of the brain. All these trachæa afterwards continue, and terminate on the choroid. The genera which have no choroid also want the circular trachea.

In the genera which have vesicular tracheæ, like the *lamellicorn coleopteræ,* most of the *lepidopteræ* and *dipteræ,* as well as certain *orthopteræ,* like the *gryllus* and the *truxalis,* we observe considerably under the optic cone another circular trachea, but much smaller than that which surrounds the edge of the cornea. The latter turns around the optic nerve, and is surrounded itself by numerous air pouches, the use of which seems to be, to sustain the optic nerve, and to keep it in its position.

The small circular trachea is wanting in all the genera which do not present pneumatic pouches or vesicular tracheæ: as it appears, however, essential for keeping the nerve in its position, it is replaced by the fibres of the adductor muscle of the mandibles, which on separating wholly surround the optic nerve, and prevent its being put out of place. We cannot say that the muscle in its contractions can act on the nerve by compressing it; for observation proves, that, by placing the muscle in all the contractions of which it is capable, the nerve remains always in its natural position, since the contraction of the muscle is effected longitudinally only, and from front to rear; so that, whatever contraction it undergoes, it can never touch the optic nerve.

The optic nerve formed by the prolongation of the brain is, of all the nerves of the head, the largest and broadest, particularly if we measure it at the place where it spreads. It issues almost always from the lateral and upper surfaces of the brain; but its position with respect to the other nerves furnished by the brain is very variable. According to the species and position of the various parts situated in the head, it is either the third, the fourth, or the fifth, pair of nerves furnished by this organ. The optic nerve at its origin is a little cylindrical, and, directing itself laterally, enters soon after its origin into the small circular trachea when it exists; and, when it does not exist, between the filaments of the adductor muscle of the mandible, which forms a kind of circular aperture for its passage. Gradually this nerve expands, and forms a cone, which has its base on the cornea and its summit on the brain. This expansion is greater or less according to circumstances. The *libel-*

*lulæ*, the *lamellicorn coleopteræ*, most of the *lepidopteræ*, as well as the *gryllus*, the *truxalis*, and the *mantis*, present it almost the whole length of the cornea; whereas in the greater number of species in which the cornea is not spherical, this breadth is in general much less, and that in a very decided manner. It is from this expansion that a very considerable number of nervous filaments issue, which proceeding between the tracheæ furnished by the large circular trachea, form the network which we have already mentioned. These are the filaments which, traversing the choroid and its varnish, as well as the tunic of the cornea, go to form the particular retina of each facet, each penetrating into the hollow of one of these facets, in order to receive the impression of the light which they transmit to the brain. We see at the exterior of the compound eyes a black point which seems moveable, and the apparent mobility of which is owing to a cause which we shall explain when speaking of vision in general. In order to resume this description, we may observe that, according to the conformation of the compound eyes, the union of the small facets forms altogether the first membrane or transparent cornea, and that, besides, each of these facets may be considered as itself a cornea. The nervous filaments which pass through the tunic of the cornea are probably the particular retina of each facet. As to the tunic with which they are as it were surrounded, and which every where fringe the cornea, its chief use must be to diminish the impression of the luminous rays, an impression the stronger because it takes place in an immediate manner. The blackish varnish which covers the opaque membrane, situated under the tunic of the cornea, may with great probability be assimilated to the varnish of the choroid, as the membrane itself may be to the choroid. Finally, the expansion of the optic nerve applied under the choroid, must be considered with M. Cuvier as a true nervous membrane perfectly similar to that of the red-blooded animals.

After having given a general description of the compound eyes of insects, it only remains for us to describe some peculiarities of organization which different species present.

If we study the compound eye of certain *lamellicorn coleopteræ*, as, for example, that of the *nasicorn geotrupæ* and *silenæ*, we observe that its form is like a heart, and that it is divided into two in its upper part, by the portion of the cranium which supports the horn. The cornea is tolerably thick, and divided, as usual, into hexagonal facets: under this membrane we observe the mucous tunic, the optic filaments, the varnishing of the choroid, and that membrane itself, black like the tunic of the cornea.

The circular trachea exists, and the great optic nerve receives, besides an infinity of other tracheæ, several of which accompany the small optic nerves, and reach the cornea by some subtile ramifications. The roots or principal trunks of the tracheæ are placed under the optic nerve, and are attached in the first instance to the tunic with which it is covered. They derive their origin from a considerable branch adhering below the principal trachea, and surrounded rounded with other small similar tracheæ. The *geotrupæ*, flying only at sunset, present the same organization in their optic nerve with most of the *lucifugæ*. Their principal retina or great optic nerve approaches nearer to the cornea than in the species which fly about in open day. Thus the eyes of the giant *scarite* become after the death of the insect of a reddish white, and yet they are black when the insect is alive. This whitish colour is owing to an alteration in the tunic of the cornea; but as this alteration does not act upon the mucous varnish of the choroid, the latter remains black, of which we may be convinced on removing the cornea with its tunic. This appearance is also very common in an infinite number of *coleopteræ*, and even in several other families. In general the cornea is very thick in the eyes of the *coleopteræ:* a few only of the *hymenopteræ* (and in particular the *apis violacea*) present this membrane of an equal thickness. When the *coleopteræ* have vesicular tracheæ only, which takes place in almost all the *lamellicorn* insects, they want the circular trachea which generally surrounds the optic nerve. To conclude—the optic nerve is always surrounded with tracheæ even in a considerable number, and these tracheæ form most frequently several vesicles, which leave some interval between them, an interval generally very small. Nevertheless, in the species having the tunic of the cornea of a clear colour, instead of being black, we see on the exterior part of the eye a single point of the same colour corresponding to the aperture through which the optic nerve passes; or rather we distinguish several points, as we shall mention when we come to speak of the butterfly.

(To be continued.)

